# Performance of 3D Network-Structured LiFePO_4_@Li_3_V_2_(PO_4_)_3_/Carbon Nanofibers via Coaxial Electrospinning as Self-Supporting Cathode for Lithium-Ion Batteries

**DOI:** 10.3390/ma18091969

**Published:** 2025-04-26

**Authors:** Ruixia Chu, Hongtao Zhang, Wanyou Huang, Fangyuan Qiu, Yan Wang, Zhenyu Li, Xiaoyue Jin

**Affiliations:** 1 Automotive Engineering College, Shandong Jiaotong University, Jinan 250357, China; 202106@sdjtu.edu.cn (R.C.); zht13293907672@163.com (H.Z.); 202103@sdjtu.edu.cn (F.Q.); sdzbgqwangyan@163.com (Y.W.); 15306477529@163.com (Z.L.); 15834185062@163.com (X.J.); 2 Intelligent Testing and High-End Equipment of Automotive Power Systems, Shandong Province Engineering Research Center, Jinan 250357, China; 3 Jinan Engineering Research Center of Automotive Equipment and Technology, Jinan 250357, China

**Keywords:** LiFePO_4_@Li_3_V_2_(PO_4_)_3_, self-supporting cathode, coaxial electrospinning, lithium-ion batteries, carbon nanofibers, rate capability

## Abstract

Lithium-ion batteries (LIBs) with high power, high capacity, and support for fast charging are increasingly favored by consumers. As a commercial electrode material for power batteries, LiFePO_4_ was limited from further wide application due to its low conductivity and lithium-ion diffusion rate. The development of advanced architectures integrating rational conductive networks with optimized ion transport pathways represents a critical frontier in optimizing the performance of cathode materials. In this paper, a novel self-supporting cathode material (designated as LFP@LVP-CES) was synthesized through an integrated coaxial electrospinning and controlled pyrolysis strategy. This methodology directly converts LiFePO_4_, Li_3_V_2_(PO_4_)_3_, and polyacrylonitrile (PAN)) into flexible, binder-free cathodes with a hierarchical structural organization. The 3D carbon nanofiber (CNF) matrix synergistically integrates LiFePO_4_ (Li/Fe/PO_x_) and Li_3_V_2_(PO_4_)_3_ (Li/V/PO_x_) nanoparticles, where CNFs act as a conductive scaffold to enhance electron transport, while the PO_x_ polyanionic frameworks stabilize Li^+^ diffusion pathways. Morphological characterizations (SEM and TEM) revealed a 3D cross-connected carbon nanofiber matrix (diameter: 250 ± 50 nm) uniformly embedded with active material particles. Electrochemical evaluations demonstrated that the LFP@LVP-CES cathode delivers an initial specific capacity of 165 mAh·g^−1^ at 0.1 C, maintaining 80 mAh·g^−1^ at 5 C. Notably, the material exhibited exceptional rate capability and cycling stability, demonstrating a 96% capacity recovery after high-rate cycling upon returning to 0.1 C, along with 97% capacity retention over 200 cycles at 1 C. Detailed kinetic analysis through EIS revealed significantly reduced R_ct_ and increased Li^+^ diffusion. This superior electrochemical performance can be attributed to the synergistic effects between the 3D conductive network architecture and dual active materials. Compared with traditional coating processes and high-temperature calcination, the preparation of controllable electrospinning and low-temperature pyrolysis to some extent avoid the introduction of harmful substances and reduce raw material consumption and carbon emissions. This original integration strategy establishes a paradigm for designing freestanding electrode architectures through 3D structural design combined with a bimodal active material, providing critical insights for next-generation energy storage systems.

## 1. Introduction

Lithium-ion batteries (LIBs), renowned for their superior capacity characteristics, have been extensively employed in diverse applications such as portable electronics, electric vehicles, and smart grids, playing a pivotal role in advancing the global energy transition [1]. However, with the rapid advancement of global economies and technologies, the escalating demands from mobile electronic devices for enhanced power performance, extended cycle life, and improved safety of LIBs have intensified. The development of electrode materials with higher power density, prolonged operational longevity, and enhanced safety has emerged as a critical research frontier in contemporary energy storage technologies.

Cathode materials, serving as the primary lithium-ion reservoirs in LIBs, have been extensively investigated, including LiFePO_4_ [2,3], LiCoO_2_ [4,5], Li(NiCoMn)O_2_ [6,7], LiMn_2_O_4_ [8,9], and Li_4_Ti_5_O_12_ [10,11]. These studies primarily focused on their crystal structures, microstructural morphologies, and energy storage mechanisms. Among them, LiFePO_4_ (LFP) has garnered significant attention due to its exceptional safety profile, structural stability, and cost-effectiveness, making it a preferred candidate for applications in new energy vehicles and green energy storage systems [12,13]. Nevertheless, the inherently low electronic conductivity and sluggish lithium-ion diffusion kinetics of LFP severely restrict its fast-charging capabilities, particularly under a high rate of discharge, where capacity retention becomes significantly compromised. These limitations primarily stem from increased electron transport resistance, which exacerbates electrochemical polarization and elevates internal resistance.

Therefore, it has become a pressing priority to address the critical scientific challenge of enhancing the electronic conductivity and lithium-ion diffusion kinetics of LFP while maintaining its excellent cycling stability. Current strategies encompass microstructural engineering [14,15,16], such as nanostructuring, porous architecture design, crystallographic orientation control, and single-crystal synthesis; surface modification/coating with carbon-based materials, metal oxides, conductive polymers, or hybrid cathode composites [17,18]; ion doping (e.g., Mg^2+^, Co^2+^, Fe^2+^, F^−^, S^2−^) [19,20,21]; electrode/electrolyte interface optimization [22,23]; and advanced synthesis techniques. Recent advancements highlight the efficacy of integrated approaches [24,25] in synergistically improving cycling stability and electrochemical kinetics. For instance, Liu et al. [26]. utilized microbial residues through acid–base pretreatment to convert them into a nitrogen (N)-, fluorine (F)-, and phosphorus (P)-enriched biomass carbon source (NFPC). By employing wet ball milling and high-temperature sintering techniques, the NFPC was coated onto LFP to prepare a three-dimensional porous-structured LFP@NFPC composite material. Electrochemical tests demonstrated that the LFP@NFPC||graphite full battery exhibited a discharge specific capacity of 161.2 mAh·g^−1^ at 1 C, and the LFP@NFPC-2||graphite full battery maintained a capacity retention rate of 82.8% after 1000 cycles at 10 C. These results proved that the N, F, and P co-doping and the construction of the three-dimensional porous structure effectively improved the electronic conductivity and lithium-ion diffusion kinetics of LFP, significantly enhancing battery performance. Zhong et al. [27] synthesized N/S-doped carbon-coated LFP through a one-step ball milling combined with high-temperature heat treatment method, where zein was used as both the carbon source and heteroatom donor. This strategy synergistically optimized material performance by enhancing conductivity through carbon coating, introducing defects/active sites via N/S doping in the carbon network, and controlling particle size (the carbon layer inhibited grain growth to 20–70 nm). Electrochemical tests demonstrated that NS/C15@LFP achieved an initial discharge capacity of 155.97 mAh·g^−1^ at 0.1 C, retained 84.06 mAh·g^−1^ even at 20 C, and maintained 87% capacity retention at 1 C and 89% at 5 C after 500 cycles. The integrated approach effectively increased lithium-ion diffusion rate (D (Li^+^) reaching 3.13 × 10^−9^ cm^2^·s^−1^) and reduced charge transfer resistance (R_ct_ = 98.32 Ω), significantly improving the cycling stability and electrochemical reaction kinetics of LFP.

Li_3_V_2_(PO_4_)_3_ (LVP) has attracted extensive research attention in recent years due to its high ion diffusion coefficient, elevated discharge plateau voltage, and superior rate capability, which collectively contribute to its exceptional energy density. Additionally, LVP was demonstrated remarkable low-temperature performance and environmental compatibility, further solidifying its potential as a promising cathode material for advanced LIBs [28,29,30]. For instance, Chien et al. [31] prepared LFP/LVP/C (LFVP-91/C) composite cathode materials with a molar ratio of LFP:LVP = 9:1 through sol–gel (SG) and spray drying (SP) techniques combined with LVP and multiple carbon composites. This approach obviously enhanced the conductivity and lithium-ion diffusion capability of LFP. The interfacial resistance was effectively reduced by incorporation of graphene oxide (GO), and it was lowered to 41.31 Ω because of the further introduction of graphene nanosheets (GNSs) and carbon nanotube (CNT) hybrid carbon additives. The SP-LFVP-91/C/GNS + CNT composite exhibited a substantially improved lithium-ion diffusion coefficient of 1.26 × 10^−13^ cm^2^·s^−1^, along with excellent discharge capacity (160.1 mAh·g^−1^ at 0.1 C) and cycling stability (85.26% capacity retention after 500 cycles at 1 C), demonstrating enhanced performance across various charge–discharge rates. Chen et al. [32] prepared LFP·LVP/C composite cathode materials to improve the electrochemical performance of LFP. During the preparation process, spray drying facilitated the formation of spherical structures to reduce particle aggregation, while calcination in a carbon reduction atmosphere promoted the generation of porous structures. The synergistic effect of these two methods significantly enhanced conductivity and lithium-ion diffusion capability. For example, the LFP·LVP/C-2 composite exhibited a specific capacity of 170 mAh·g^−1^ at 0.2 C, maintained over 90% capacity retention after 100 cycles at 1 C, and delivered a discharge capacity of 70 mAh·g^−1^ even at 10 C, demonstrating excellent electrochemical performance.

Conventional electrode materials synthesis approaches (e.g., wet ball milling, sol–gel methods) typically require high-temperature calcination processes, which could result in excessive energy consumption and potential crystal structural degradation (e.g., lattice defects or grain coarsening). Additionally, conductive additives and binders used in complex traditional slurry-coating processes limit the intrinsic performance of active materials. The poor connection between components and current collectors results in high impedance and easy detachment of active materials during cycling. Furthermore, the use of toxic solvents (e.g., N-methylpyrrolidone, NMP) and high-temperature calcination (>800 °C) further exacerbate environmental contamination risks, failing to align high energy density with sustainable manufacturing practices. These limitations highlight the urgent need for innovative electrode architectures to break these bottlenecks.

Concurrently, studies have demonstrated that three-dimensional (3D) networks composed of carbon nanofibers (CNFs) can efficaciously enhance electronic/ionic transport efficiency and mitigate volumetric strain during cycling [33,34]. The electrospinning technique, combined with thermal processing, enables the fabrication of 3D CNF architectures with tunable micro/nano-structures, which are highly adaptable for synthesizing multifunctional composites. When applied to LIB electrodes, these CNF-based frameworks markedly improve charge/discharge kinetics, rate capability, and cycling stability [35,36], while addressing diverse application requirements such as high-energy-density storage and flexible electronics [37,38]. Zhuang et al. [39]. prepared 3D free-standing CNFs via the electrospinning method, in situ modified with lithiophilic metal particles (Sn, Fe, and Co) to form CNF/Me composite materials (Me=Sn, Fe, Co). Among them, the CNF/Sn-Li composite electrode demonstrated outstanding electrochemical performance when paired with a commercial LFP cathode: At 1 C (1 C = 170 mAh·g^−1^), the LFP//CNFs/Sn-Li full cell exhibited an initial discharge specific capacity of 139 mAh·g^−1^, which remained at 146 mAh·g^−1^ after 400 cycles with a stable Coulombic efficiency of approximately 99%, even under high areal mass loading (11 mg·cm^−2^). Moreover, it maintained a reversible capacity of 131 mAh·g^−1^ and Coulombic efficiency of 95.4%, much superior to the control group using a bare Li anode (67.6 mAh·g^−1^, 93.3%). Liu et al. [40] successfully fabricated 3D LFP@reduced graphene oxide/carbon nanofibers (LFP@rGO/CNFs) as flexible cathodes for LIBs through the electrospinning technique combined with subsequent pyrolysis. The composite material features a uniform attachment of rGO sheets onto carbon nanofibers and a homogeneous distribution of LFP nanoparticles within the carbon fibers, forming a 3D continuously conductive network. Electrochemical tests demonstrated an initial capacity of 167 mAh·g^−1^ at 0.5 C, maintaining 150 mAh·g^−1^ reversible capacity after 200 cycles at 1 C with 98.9% retention. The galvanostatic intermittent titration technique (GITT) revealed a high lithium-ion diffusion coefficient of 8.82 × 10^−12^ cm^2^·s^−1^, while the charge transfer resistance (20.956 Ω) was clearly lower than that of the control group without rGO addition (126.798 Ω), indicating superior rate capability and structural stability. Nonetheless, current research on self-supporting LFP-based cathodes lacks comprehensive exploration of dual-compound composites (e.g., LFP/LVP), particularly regarding synergistic mechanisms.

To sum up, the design of a LFP-based cathode material synergistically integrated with LVP and anchored on a cross-connected 3D CNF architecture as a self-supporting substrate offers an innovative fabrication strategy for advancing high-performance cathode materials.

In this study, a novel 3D LFP@LVP/CNF (LFP@LVP-CES) self-supporting cathode was synthesized through a coaxial electrospinning and controlled pyrolysis strategy. The cathode materials integrate LVP composite optimization, carbon nanofiber encapsulation, and 3D conductive network architecture, synergistically heightening power density and cycling stability. Firstly, a cross-connected 3D CNF framework that tightly encapsulates active materials (LFP and LVP) was fabricated by the electrospinning technique, significantly enlarging the electrode–electrolyte inter-facial contact area. This architecture shortens Li^+^ diffusion pathways, accelerates ion transport kinetics, and mitigates volume expansion during cycling, thereby improving rate capability and structural integrity [41,42]. Secondly, high-conductivity LVP and high-stability LFP further enabled complementary integration within the nanofibers through a coaxial hybrid electrospinning process, fostering synergistic charge transfer enhancement and ion diffusion dynamics. At the atomic level, the olivine-structured LFP stabilizes the crystal lattice through its (PO_4_)^3−^ polyanionic framework and facilitates one-dimensional Li^+^ diffusion mainly along the [010] channel, while the NASICON-structured LVP provides a three-dimensional open framework with interconnected interstitial sites, enabling higher Li^+^ mobility and structural stability. At the composite level, the 3D CNF matrix acts as a conductive scaffold, bridging isolated LFP and LVP particles to form continuous electron transport pathways. This hierarchical design minimizes interfacial resistance between active materials and the CNFs, resulting in superior electrochemical performance in the composite. This dual-strategy interaction not only elevates specific capacity but also stabilizes long-term cycling performance. Finally, the mechanical reinforcement provided by the 3D CNF network and active particles endows the electrode with intrinsic flexibility, self-supporting functionality, and robust mechanical strength, eliminating reliance on metallic current collectors. Furthermore, unlike conventional carbon-coated LFP cathodes, which rely on slurry-coating processes involving toxic solvents (e.g., N-methylpyrrolidone, NMP) and high-temperature calcination (>800 °C), the self-supporting LFP@LVP-CES cathode is fabricated via controlled electrospinning and low-temperature pyrolysis (680 °C) of polyacrylonitrile (PAN)-derived CNFs. This approach significantly reduces energy consumption, raw material usage, and carbon emissions while avoiding NMP-related hazards [43], thereby contributing to sustainable manufacturing practices in the battery industry.

This innovation reduces raw material costs, conserves mineral resources, and improves gravimetric energy density, offering a transformative pathway for developing high-performance, low-cost LIBs.

## 2. Experimental Section

### 2.1. Synthesis of LFP@LVP/CNFs

In this study, a 3D LFP@LVP/carbon nanofiber (CNF) composite was synthesized via coaxial hybrid electrospinning. The composite was synthesized via coaxial electrospinning, a well-established technique for fabricating hierarchical fiber architectures [44]. Commercial-grade LFP (magnetic impurity content: ppm level, Canrd, Dongguan, China) and LVP (magnetic impurity content: ppm level, Canrd, Dongguan, China) were employed as active materials, with polyacrylonitrile (PAN, Mw~15 w, Aladdin, Shanghai, China) serving as the polymeric matrix and dimethylformamide (DMF, AR, Macklin, Shanghai, China) as the solvent. All reagents were procured from qualified manufacturers to ensure consistency and reproducibility.

The composite material was synthesized through the following procedures: Firstly, 0.629 g of PAN was completely dissolved in 6 mL of DMF under magnetic stirring at 80 °C for 3 h to obtain a homogeneous transparent solution. Subsequently, 0.5 g of LFP was uniformly dispersed into the solution and magnetically stirred for 2 h to form a homogeneous LFP spinning precursor. The LVP spinning precursor was prepared using the same method. Both precursors were co-extruded through a dual-channel coaxial spinneret to generate composite nanofibers.

**Figure 1:**
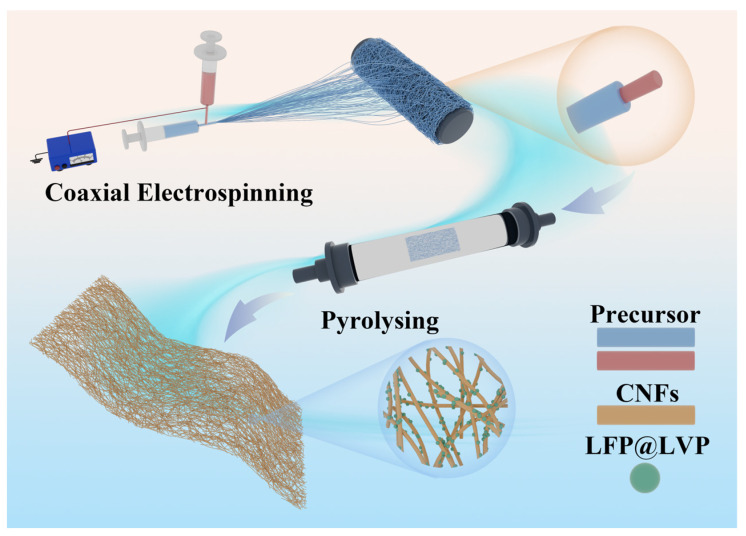
Schematic illustration of the fabrication for LiFePO_4_/Li_3_V_2_(PO_4_)_3_@CNFs via coaxial electrospinning and thermal treatment.

The electrospun fibers formed a white organic membrane composed of interwoven nanofibers, which was vacuum-dried at 150 °C for 24 h, cut into 10 cm × 5 cm strips, and subjected to high-temperature thermal treatment. The thermal process involved two stages: pre-oxidation at 250 °C in air for 3 h to remove residual solvents and enhance structural integrity, followed by carbonization at 680 °C under a nitrogen atmosphere for 8 h. During carbonization, PAN decomposed into a carbonaceous matrix, which interpenetrated with LFP and LVP particles to form a 3D integrated electrode structure. The entire fabrication workflow is illustrated in Figure 1.

To investigate the electrochemical performance of the synthesized LFP@LVP/CNF composite as a cathode for LIBs, four control samples were prepared: electrospun fibers with LFP and LVP as the sole active materials, denoted as LFP-ES and LVP-ES, respectively, composite fibers fabricated via coaxial hybrid electrospinning of LFP and LVP (denoted as LFP@LVP-CES), and a composite electrode prepared through conventional coating with a mixture of LFP and LVP in the same ratio (denoted as LFP@LVP-Coating).

### 2.2. Materials Characterizations

The crystalline phases of the samples were analyzed by X-ray diffraction (XRD, SmartLab, Rigaku, Tokyo, Japan) with a scanning angle range of 10° to 90° to determine the crystallographic composition and crystallinity. Scanning electron microscopy (SEM, MERLIN Compact, Carl Zeiss, Oberkochen, Germany) was employed to investigate the microstructural morphology, focusing on the interpenetrating network architecture and the morphology of blended fibers fabricated via the coaxial electrospinning process. Thermogravimetric analysis (TGA, Q5000, TA Instruments, New Castle, DE, USA) was conducted to evaluate thermal stability and active material content under an air atmosphere, with a heating rate of 10 °C·min^−1^ from room temperature to 800 °C. Transmission electron microscopy (TEM, JEM-2100F, JEOL Ltd., Akishima, Japan) was further utilized to observe lattice structures and elemental distribution at atomic resolution.

### 2.3. Electrochemical Measurement

Electrochemical characterization was performed using CR2016 coin-type cells. For each sample group (LFP-ES, LVP-ES, LFP@LVP-CES, and LFP@LVP-Coating), 10–12 cells were independently assembled to ensure data robustness.

The assembly process of the coin-type half-cells is shown as follows: First, the self-supporting cathode was cut into circular disks (13 mm in diameter) using a manual punch (MRX-CP60, Mingruixiang, Shenzhen, China). The cells were assembled in an argon-filled glove box (MKSS10045, Mikrouna, Shanghai, China, H_2_O/O_2_ ≤ 0.01 ppm). Components, including the cathode shell, self-supporting cathode disk, separator (Celgard 2400, Dongguan Kelude New Energy Technology Co., Ltd., Dongguan, China), lithium foil, and spacer, were stacked sequentially. The electrolyte (conductivity: 10.76 ms·cm^−1^; density: 1.2276 g·cm^−3^; free acid (calculated as HF) ≤ 50 ppm, Dongguan Kelude New Energy Technology Co., Ltd.) consisted of 1 M lithium hexafluorophosphate (LiPF_6_) dissolved in a 1:1:1 (*v*/*v*) mixture of ethylene carbonate (EC), diethyl carbonate (DEC), and dimethyl carbonate (DMC). To enhance electrochemical stability, 1.0 vol% vinylene carbonate (VC) was added. A stepwise infiltration process was employed, injecting 20 μL and 40 μL of electrolyte sequentially to ensure thorough wetting of the electrode and separator. Finally, the cell casing was sealed using a crimping machine (MRX-SF120, Mingruixiang, Shenzhen, China), and the open-circuit voltage was preliminarily measured with a multimeter to confirm the assembly quality.

Galvanostatic charge–discharge (GCD) tests were conducted on a LAND CT2001A testing system (Land Electronics, Wuhan, China) at ambient temperatures of 10–15 °C within a voltage range of 2.5–4.2 V (vs. Li/Li^+^). Cyclic voltammetry (CV) and electrochemical impedance spectroscopy (EIS) measurements were performed using an Autolab PGSTAT302N electrochemical workstation (Metrohm AG, Herisau, Switzerland). CV scans were carried out at a sweep rate of 0.1 mV·s^−1^ between 2.5 and 4.5 V (vs. Li/Li^+^), while EIS was measured with an amplitude of 10 mV over a frequency range of 10 kHz to 0.01 Hz.

For all prepared samples and assembled cells, the synthesis technology, assembly process, characterization, and electrochemical measurement were carried out under the same environmental conditions and employing the same equipment to ensure the repeatability of the research process and the stability of the research results.

## 3. Results and Discussion

**Figure 2:**
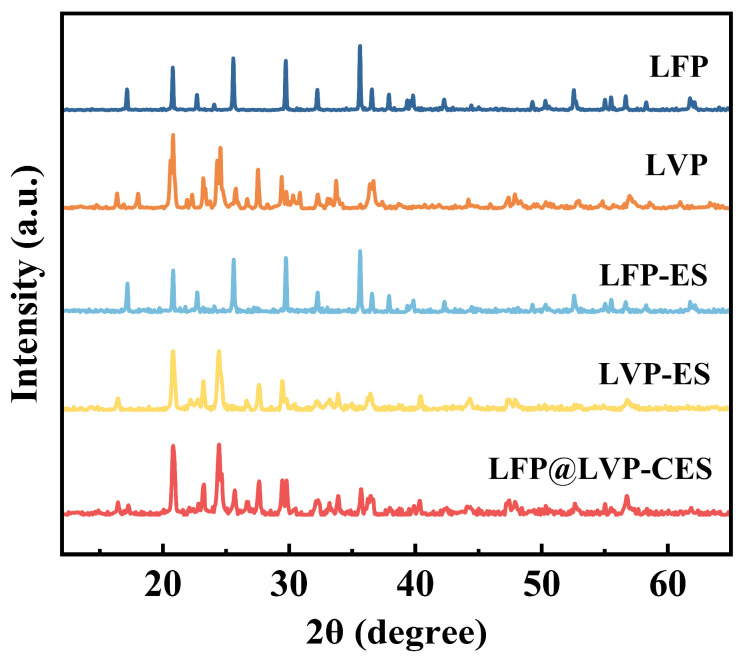
XRD patterns of LFP, LVP, LFP-ES, LVP-ES, and LFP@LVP-CES.

Figure 2 displays the XRD patterns of LFP, LVP, LFP-ES, LVP-ES, and LFP@LVP-CES. The diffraction peaks of LFP powder align precisely with the characteristic peaks of standard LFP (PDF#97-026-0570), confirming its typical olivine-type crystal structure. Similarly, the diffraction peaks of LVP powder correspond well to the reference pattern of LVP (PDF#97-016-8267), validating its monoclinic phase composition. For LFP-ES and LVP-ES, the peak positions show no significant shifts compared to the pristine powders, indicating that the high-temperature carbonization process primarily forms a carbon layer on the composite surface or within the matrix without altering the bulk crystal structure. This structural stability is critical for maintaining smooth lithium-ion intercalation/deintercalation kinetics and ensuring robust electrochemical performance. In the case of LFP@LVP-CES, the XRD pattern exhibits the simultaneous characteristic peaks of both LFP and LVP, with peak positions and shapes closely matching those of LFP-ES and LVP-ES, confirming the successful fabrication of a dual-active material composite (LFP and LVP) embedded within a carbon nanofiber matrix via coaxial hybrid electrospinning and controlled pyrolysis strategy and further highlighting the compatibility and structural integrity of the synthesis strategy.

**Figure 3:**
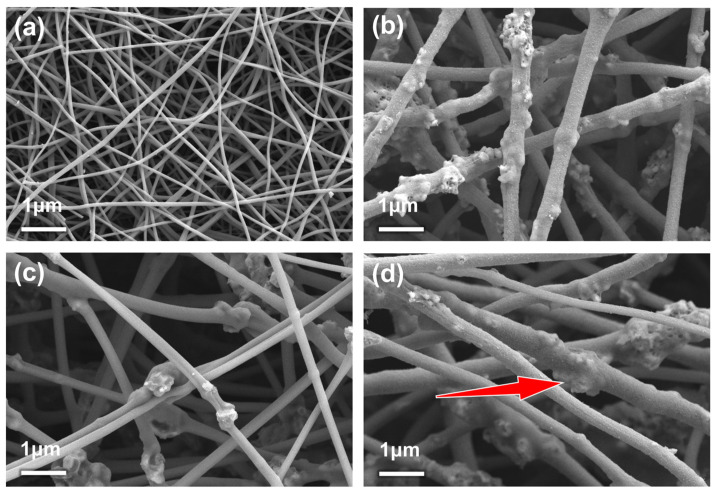
SEM images of (**a**) PAN-ES, (**b**) LFP-ES, (**c**) LVP-ES, and (**d**) LFP@LVP-CES. (The red arrow indicates LFP and LVP particles embedded within the CNF matrix).

Figure 3 presents the SEM images of the samples. As shown in Figure 3a, PAN-ES (Pure PAN electrospun fibers) exhibits an interwoven carbon nanofiber network with fiber diameters ranging from 250 to 300 nm. The interconnected fibers form capillary channels that facilitate rapid electrolyte infiltration and promotes Li^+^ diffusion. Additionally, sufficient void space between fibers ensures robust structural stability and flexibility. This self-supporting framework effectively accommodates volume changes during charge/discharge cycles, maintaining structural integrity and stabilizing electrochemical performance. As shown in Figure 3b–d, the microstructures of LFP-ES, LVP-ES, and LFP@LVP-CES are fundamentally consistent. A CNF network is utilized as the substrate for all three samples, successfully loading active material particles in a hybrid surface-attached and encapsulated configuration. Notably, LFP@LVP-CES (Figure 3d) achieves a homogeneous mixture of LFP and LVP particles. The majority of active particles are uniformly embedded within the CNF matrix, while a small fraction, which are agglomerated and exceed the fiber diameter in size, adhere to the fiber surfaces. These are coated by a thin carbon layer, directly in contact with the electrolyte, shortening Li^+^ diffusion pathways, reducing ion transport resistance, and enhancing the composite’s electronic conductivity, thereby improving the electrode’s rate capability.

**Figure 4:**
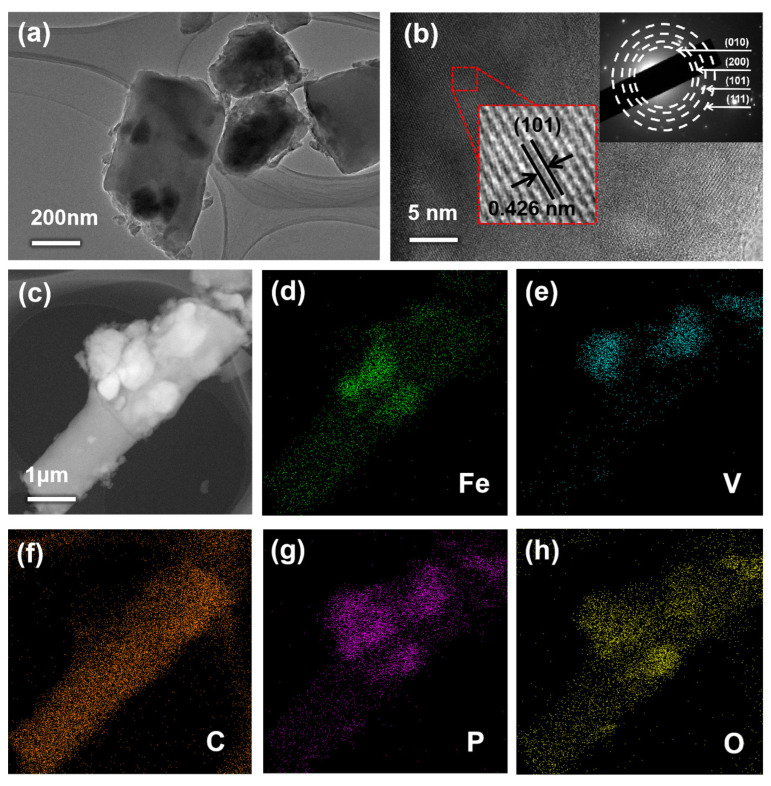
(**a**) TEM image of LFP@LVP-CES, (**b**) HR-TEM image of LFP@LVP-CES, (**c**–**h**) EDS elemental mapping images.

Further TEM characterization explores the refined microstructure of LFP@LVP-CES (Figure 4a). The dual-active particles are encapsulated within the CNF matrix, forming stable interfacial structures. This encapsulation minimizes direct contact between active materials and the electrolyte, thereby suppressing parasitic side reactions, while simultaneously mitigating volume changes during charge/discharge cycles. These features ensure the long-term structural integrity and cycling stability of the electrode. The HR-TEM image (Figure 4b) displays distinct lattice fringes corresponding to the (101) crystallographic plane of LFP, confirming that the high-temperature carbonization process preserves the crystalline structure of the active material. The selected-area electron diffraction (SAED) pattern further corroborates the presence of highly crystalline regions through discrete diffraction spots, validating structural coherence and phase purity. The EDS elemental mapping of LFP@LVP-CES (Figure 4c–h) visually reveals the spatial distribution of key elements within the composite. The Fe (green) and V (cyan) signals correspond to the locations of LFP and LVP active materials, respectively. C (orange) is uniformly distributed across the entire region, confirming the presence of the interconnected carbon nanofiber (CNF) network. P (purple) and O (yellow) signals align with the positions of Fe and V, further confirming the homogeneous mixing of LFP and LVP within the matrix. These results conclusively validate the 3D structure of LFP/LVP hybrid embedded in the CNF framework, where both active materials are uniformly dispersed and stabilized.

**Figure 5:**
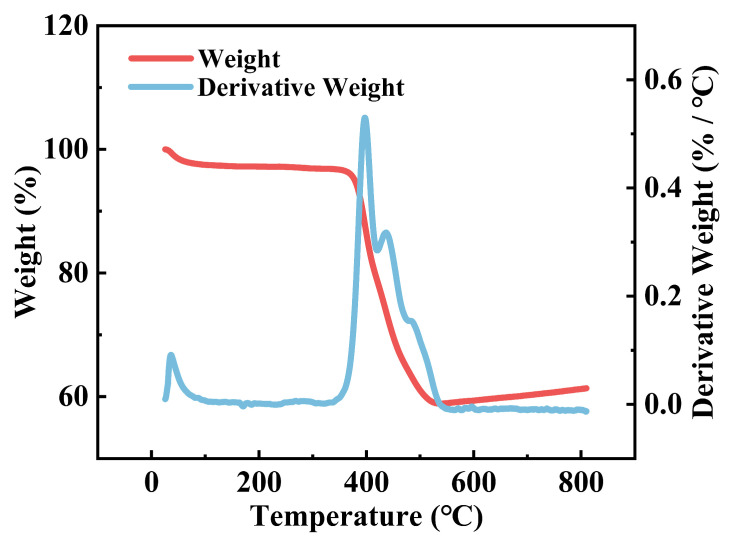
TG and TGA curves of LFP@LVP-CES.

To determine the effective active material content in the LFP@LVP-CES electrode, thermogravimetric analysis (TGA) was performed with a heating ramp of 10 °C·min^−1^ from 25 to 800 °C in air. Figure 5 shows the TG (red curve) and DTG (blue curve) profiles of the LFP@LVP-CES composite. During the low-temperature stage (room temperature to ~200 °C), a minor mass loss (~2–3 wt%) is observed, attributed to the removal of adsorbed moisture and volatile impurities. In the intermediate-temperature stage (~200–400 °C), a rapid mass reduction (~25 wt%) occurs due to the oxidation of CNFs, accompanied by a prominent peak in the DTG curve. At high temperatures (>400 °C), the mass loss rate slows as residual carbonaceous components are fully oxidized, stabilizing at ~60 wt% of the initial mass. After the temperature exceeds 600 °C, there is a slight increase in mass, which may be due to further oxidation of iron and vanadium. This residual mass corresponds to the total active material content (LFP + LVP), confirming that ~60 wt% of the composite comprises electrochemically active phases.

**Figure 6:**
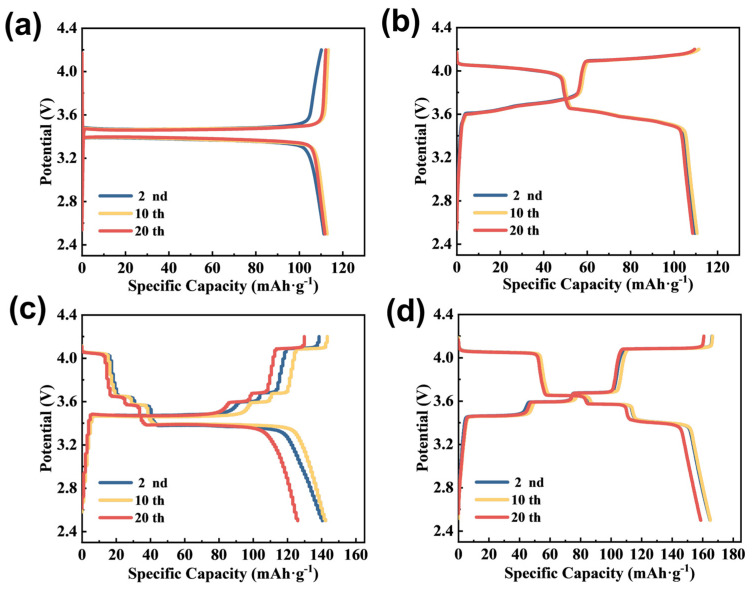
Charge–discharge curves of (**a**) LFP-ES, (**b**) LVP-ES, (**c**) LFP@LVP-Coating, and (**d**) LFP@LVP-CES.

To evaluate the electrochemical properties of the LFP@LVP-CES composite as a LIB cathode, galvanostatic charge–discharge (GCD) and cyclic voltammetry (CV) tests were conducted. GCD tests were performed at a rate of 0.1 C within a voltage range of 2.5–4.2 V. As shown in Figure 6a, the charge–discharge curves of LFP-ES exhibit typical voltage plateaus near 3.5 V and 3.4 V, corresponding to Li^+^ intercalation/deintercalation in LFP, with minimal polarization and high reversibility. For LVP-ES (Figure 6b), multiple voltage plateaus between 3.5 and 4.0 V are observed, consistent with the multi-step redox reactions of LVP, displaying comparable discharge capacity and reversibility to LFP-ES. The LFP@LVP-Coating composite (Figure 6c) presents distinct voltage plateaus for both Fe^2+^/Fe^3+^ and V^3+^/V^4+^ redox processes. However, from the second to the twentieth cycle, the charge–discharge curves exhibit poor reproducibility, with serious capacity fading in the initial voltage plateaus, leading to a marked decline in specific discharge capacity. This instability is attributed to the simple mixing and coating process, which fails to leverage the synergistic effects of dual-active materials (LFP and LVP). In contrast, the LFP@LVP-CES composite (Figure 6d) demonstrates stable voltage plateaus for both LFP and LVP, minimal electrochemical polarization, and excellent reversibility. At 0.1 C, the 20th-cycle discharge capacity of LFP@LVP-CES is 24% higher than that of LFP@LVP-Coating. This enhancement arises from the 3D conductive network formed by coaxial electrospinning, which integrates LFP and LVP within a carbon nanofiber matrix. The synergistic interaction between LFP and LVP suppresses “dead lithium” formation and ensures efficient ion/electron transport, thereby improving structural stability and capacity retention.

**Figure 7:**
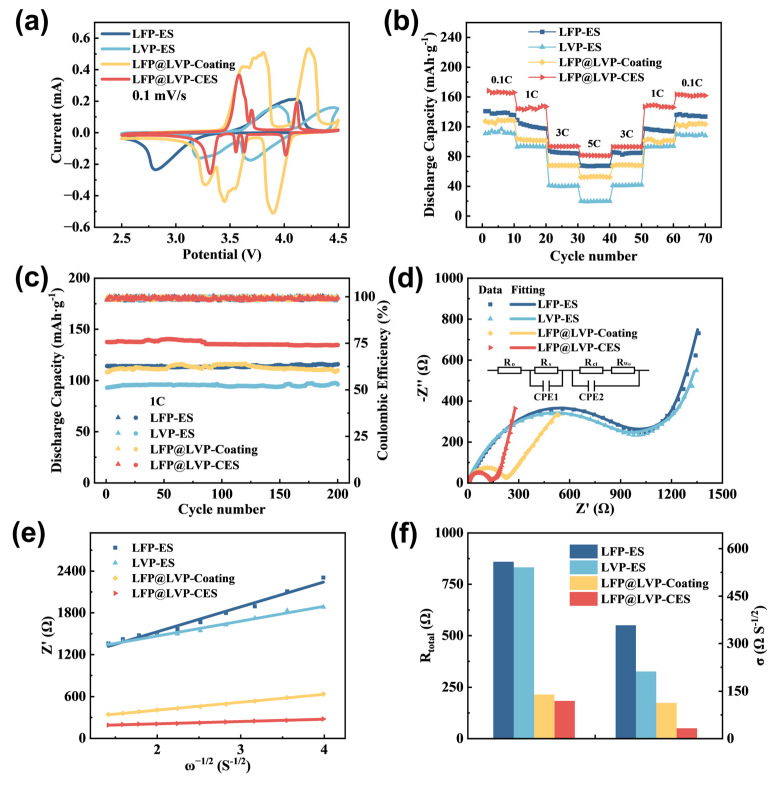
(**a**) Cyclic voltammetry (CV) curves; (**b**) rate capability curves; (**c**) cycling performance; (**d**,**e**) EIS analysis curves; (**f**) calculated total internal resistance and Warburg coefficient.

The CV curves of the four prepared samples are shown in Figure 7a. For LFP-ES and LVP-ES, the oxidation/reduction peak potentials deviate from the standard Li^+^ intercalation/deintercalation potentials of pristine LFP and LVP. This discrepancy arises from the 4.5 V adjusted upper cutoff potential used in CV testing for the LFP/LVP composite system. At this elevated potential, the ion/electron transport kinetics of the active materials are constrained, leading to a pronounced shift in oxidation peaks (delithiation) to higher voltages and reduction peaks (lithiation) to lower voltages. Consequently, the peak potential separation (ΔEp) increases markedly, indicating severe electrochemical polarization. Notably, for LFP, exposure to 4.5 V may induce irreversible phase transitions (e.g., partial conversion to Fe_2_O_3_ or Li_3_PO_4_), weakening or eliminating the primary oxidation peak at 3.4 V and introducing broad peaks at higher potentials (4.0–4.3 V). On the contrary, LFP@LVP-Coating and LFP@LVP-CES exhibit distinct oxidation/reduction peaks corresponding to both LFP and LVP. However, LFP@LVP-Coating shows slight peak shifts (oxidation to higher voltages and reduction to lower voltages) due to interfacial inhomogeneity from the conventional coating process, aligning with its charge–discharge voltage plateaus. LFP@LVP-CES demonstrates symmetric oxidation/reduction peaks near 3.6/3.4 V (Fe^2+^/Fe^3+^ redox couple), two pairs of peaks in the 3.5–3.7 V range, and peaks at 4.0/4.2 V (V^3+^/V^4+^ multi-step reactions). A peak overlap near 3.6 V is observed due to the synergistic interaction between LFP and LVP. All peak potentials align with the charge–discharge plateaus, confirming that despite the higher cutoff potential, LFP@LVP-CES exhibits superior polarization property and redox reversibility compared to single-active-material electrodes (LFP-ES or LVP-ES). These results validate the efficacy of the dual-active-material hybridization strategy within the 3D carbon nanofiber framework, which optimizes electrochemical performance for high-voltage LIB applications. The polyanionic PO_x_ frameworks in LVP suppress oxygen evolution at high voltages through strong P-O covalent bonds, ensuring LFP@LVP-CES structural stability. Compared to the single-electron Fe^2+^/Fe^3+^ redox reaction in LFP, the multi-electron V^3+^/V^4+^ redox activity in LVP enhances the overall specific capacity of the composite. Additionally, the moderate hybridization between vanadium’s 3d orbital and oxygen’s 2p orbital optimizes the band alignment with lithium chemical potential. Combined with the high conductivity of the carbon fiber matrix, this synergy ensures robust performance under high-voltage conditions.

According to additional electrochemical tests, the LFP@LVP-CES composite was confirmed to exhibit superior rate capability, cycling stability, and impedance characteristics compared to other samples. As shown in Figure 7b, the LFP@LVP-CES composite achieved a specific capacity of ~165 mAh·g^−1^ at 0.1 C, retaining ~144 mAh·g^−1^ at 1 C (87% of the 0.1 C capacity) and significantly outperforming single-active-material electrodes (LFP-ES: ~135/120 mAh·g^−1^, LVP-ES: ~115/93 mAh·g^−1^ at 0.1/1 C). Even at 5 C, the specific capacity of LFP@LVP-CES is maintained at 80 mAh·g^−1^ and recovers to 160 mAh·g^−1^ (96% of the initial value) upon returning to 0.1 C, highlighting excellent rate resilience and fast-charging capability. This enhancement originated from the synergistic interaction between LFP and LVP in lithium-ion intercalation/deintercalation kinetics and the 3D conductive carbon nanofiber (CNF) network, which ensured rapid electron/ion transport. Figure 7c illustrates the cycling stability of the samples at 1 C. The LFP@LVP-CES composite exhibited the highest initial discharge capacity (137 mAh·g^−1^) and retained 134 mAh·g^−1^ after 200 cycles (97% capacity retention), with Coulombic efficiency approaching 100%. A counter feature of the LFP@LVP-Coating electrode, however, shows capacity decay due to structural instability inherent in conventional slurry-coating methods. The 3D CNF framework in LFP@LVP-CES effectively mitigated volume expansion, suppressed active material pulverization, and maintained electrical continuity during prolonged cycling.

To elucidate the impact of electrode micro-architecture on electrochemical performance, EIS was employed to analyze interfacial reaction kinetics. The Nyquist plots of all samples (Figure 7d) feature a semicircle in the high-to-medium frequency region, corresponding to the charge-transfer resistance (R_ct_), and a sloped line in the low-frequency region, associated with Warburg impedance (WS) linked to Li^+^ diffusion. The equivalent circuit (inset in Figure 7d) comprises RS (electrolyte resistance), R_ct_, CPE1, and CPE2 (constant phase elements for double-layer capacitance). The EIS spectra revealed that LFP@LVP-CES exhibits the lowest R_ct_ and W_S_, indicating enhanced charge-transfer kinetics and faster Li^+^ diffusion. This is attributed to the 3D conductive carbon nanofiber network, which provides interconnected pathways for electron transport and shortens ion diffusion distances. The Warburg coefficient (σ), inversely proportional to Li^+^ diffusion kinetics, is derived from the slope of the real impedance (Z’) versus the inverse square root of angular frequency (ω^−1/2^) in the low-frequency region (Figure 7e). The coefficient is calculated [40] according to Equation (1):

(1) $$R=R_{S}+R_{\mathrm{ct}}+\sigma \omega^{-1/2}$$

where R is the total resistance of the electrode (Ω), R_S_ represents the resistance associated with both the electrode and electrolyte (Ω), R_ct_ denotes the charge transfer resistance at the cathode/electrolyte interface (Ω), and σ is the Warburg coefficient inversely correlated with D (Li^+^), corresponding to the slope of the real impedance versus the inverse square root of angular frequency in the low-frequency region.

According to calculation results (Figure 7f), the R value of LFP@LVP-CES (183.71 Ω) is evidently lower than that of conventional single LFP electrode material (859.5 Ω). Simultaneously, the Warburg coefficient of LFP@LVP-CES (33 Ω·S^−1/2^) demonstrated a marked reduction compared to other single-active-material electrodes or traditional LFP/LVP slurry-coating electrodes, further confirming its superior ionic diffusion coefficient. These findings conclusively demonstrate the favorable impedance characteristics of the prepared LFP@LVP-CES composite. The incorporation of LFP into carbon nanofiber networks through electrospinning, combined with the controlled blending of LVP, effectively reduces the overall impedance of the composite material, minimizes lithium-ion diffusion resistance, and enhances diffusion kinetics. This mechanistic understanding provides substantial evidence supporting the exceptional rate capability of the composite electrode.

## 4. Conclusions

In this study, a 3D LFP@LVP/CNF (designated as LFP@LVP-CES) self-supporting composite cathode was successfully synthesized via coaxial hybrid electrospinning combined with low-temperature thermal treatment (680 °C). Structural and electrochemical characterizations comprehensively validate the superior performance of this composite. SEM and TEM characterizations demonstrate a multi-scale architecture, with active LFP and LVP nanoparticles homogenously dispersed in a three-dimensional interconnected carbon nanofiber network (Fiber diameter: 250 ± 50 nm). The CNF framework not only provides a continuous conductive network but also encapsulates the active materials, minimizing particle agglomeration and ensuring intimate contact between LFP and LVP. EDS mapping further confirms the homogeneous spatial distribution of Fe, V, P, and C elements, corroborating the structural integrity of the dual-active-material composite. TGA demonstrates that the composite retains ~60 wt% electrochemically active phases (LFP + LVP), ensuring high-capacity contribution. EIS reveals significantly reduced total electrode resistance (R = 183.71 Ω for LFP@LVP-CES vs. 859.5 Ω for conventional LFP electrodes) and enhanced Li^+^ diffusion kinetics (σ = 33 Ω·S^−1/2^), attributed to the 3D conductive CNF network that bridges isolated particles and shortens ion transport pathways. The composite exhibited excellent rate capability, achieving a specific capacity of 165 mAh·g^−1^ at 0.1 C and retaining 80 mAh·g^−1^ at 5 C, alongside long-term cycling stability with 97% capacity retention after 200 cycles at 1 C.

As shown in Table 1, the self-supporting LFP@LVP-CES composite cathode outperforms the present works in synthesis methodology, electrode architecture, and electrochemical performance. These superior electrochemical properties stem from the material’s optimized charge-transfer kinetics, reduced polarization, and enhanced Li^+^ diffusion dynamics. First, the 3D conductive CNF network constructed via electrospinning provides a continuous pathway for rapid electron/ion transport, improves electrolyte infiltration, and mitigates volume expansion during cycling, thereby enhancing structural stability and reaction kinetics. Second, the synergistic interaction between the high stability of LFP and the superior conductivity of LVP collectively boosts the composite’s specific capacity. Third, the tight encapsulation of dual active materials within the CNF matrix stabilizes the electrode/electrolyte interface, reduces interfacial resistance, and improves electronic conductivity, leading to exceptional rate capability. The coaxial electrospinning technique enables precise integration of active materials with the CNF framework, overcoming interfacial contact limitations inherent in conventional slurry-coating methods. This innovative approach offers a promising strategy for advancing LFP-based cathodes in applications such as electric vehicles and large-scale energy storage systems, particularly for flexible devices requiring high energy density and mechanical robustness.

Additionally, the self-supporting design eliminates the metallic current collectors required in traditional slurry-coating processes. The low-temperature calcination process avoids lattice defects and energy waste caused by high-temperature sintering (>800 °C) in conventional sol–gel (SG) or spray-drying (SP) methods while reducing carbon emissions.

However, this study employs commercial LFP and LVP powders as active material precursors, which exhibit large particle sizes and limited advantages in specific capacity. In future research, the in situ synthesis of nanoscale LFP and LVP via the electrospinning process could be explored. This strategy could enable the dual active materials to achieve enhanced synergistic effects at the nanoscale.

## Figures and Tables

**Table 1:** LFP@LVP-CES vs. conventional cathodes: performance and synthesis advantages.

Material	Synthesis Process	Electrode Type	Voltage Range	Rate	Cycle Number	Initial Discharge Specific Capacity (mAh·g^−1^)	Capacity Retention	Ref.
LFP	Electrospinning	Self-supporting	2.4–4.3 V	0.5 C	100	92	>90%	[45]
LFP@LVP	Sol–Gel (SG)	Coating	2.4–4.3 V	1 C	100	128.4	91.70%	[31]
	Spray Drying (SP)		2.4–4.3 V	1 C	100	148.4	96.60%	[31]
			1.5–4.2 V	1 C	100	136	>90%	[32]
	Coaxial Hybrid Electrospinning and Controlled Low-Temperature Calcination Process	Self-supporting	2.5 V–4.2 V	1 C	200	134	97%	Our work

## Data Availability

The original contributions presented in this study are included in the article. Further inquiries can be directed to the corresponding author.

## References

[1] He J., Meng J., Huang Y. (2023). Challenges and Recent Progress in Fast-Charging Lithium-Ion Battery Materials. J. Power Sources.

[2] Cao M., Liu Z., Zhang X., Yang L., Xu S., Weng S., Zhang S., Li X., Li Y., Liu T. (2023). Feasibility of Prelithiation in LiFePO_4_. Adv. Funct. Mater..

[3] Tang R., Dong J., Wang C., Guan Y., Yin A., Yan K., Lu Y., Li N., Zhao G., Li B. (2025). Rate-Dependent Failure Behavior Regulation of LiFePO_4_ Cathode via Functional Interface Engineering. Adv. Funct. Mater..

[4] Yang K., Tian R.-Z., Wang Z.-Y., Zhang H.-Z., Ma Y., Shi X.-X., Song D.-W., Zhang L.-Q., Zhu L.-Y. (2023). Regulating Surface Base of LiCoO_2_ to Inhibit Side Reactions between LiCoO_2_ and Sulfide Electrolyte. Rare Met..

[5] Li J.-J., Dai Y., Zheng J.-C. (2021). Strain Engineering of Ion Migration in LiCoO_2_. Front. Phys..

[6] Hou L., Liu Q., Chen X., Yang Q., Zhang F., Mu D., Li L., Wu F., Chen R. (2024). Al & Ti Synergy Enhancing Ionic Diffusion and Stabilizing Lattice Oxygen for the High Voltage Single Crystal Ni-Rich Layered Oxide Cathode Materials. J. Power Sources.

[7] Na S., Oh R., Song J., Lee M.-J., Park K., Park G.-S. (2025). Formation Cycle Control for Enhanced Structural Stability of Ni-Rich LiNi_x_Co_y_Mn_1−x−y_O_2_ Cathodes. ACS Nano.

[8] Shang X., Liu J., Hu B., Nie P., Yang J., Zhang B., Wang Y., Zhan F., Qiu J. (2022). CNT-Strung LiMn_2_O_4_ for Lithium Extraction with High Selectivity and Stability. Small Methods.

[9] Li F., Jiao Y., Yang S., Mao W., Tao Q., Bai C., He E., Li L., Ye T., Li Y. (2024). Electrochemical Activation Inducing Rocksalt-to-Spinel Transformation for Prolonged Service Life of LiMn_2_O_4_ Cathodes. Small.

[10] Cáceres-Murillo J., Díaz-Carrasco P., Kuhn A., Rodríguez-Castellón E., García-Alvarado F. (2024). Improvement of the Rate Capability of the Li_4_Ti_5_O_12_ Anode Material by Modification of the Surface Composition with Lithium Polysulfide. J. Alloys Compd..

[11] Wu X., Liang X., Zhang X., Lan L., Li S., Gai Q. (2021). Structural Evolution of Plasma Sprayed Amorphous Li_4_Ti_5_O_12_ Electrode and Ceramic/Polymer Composite Electrolyte during Electrochemical Cycle of Quasi-Solid-State Lithium Battery. J. Adv. Ceram..

[12] Manthiram A. (2017). An Outlook on Lithium Ion Battery Technology. ACS Cent. Sci..

[13] Manthiram A., Goodenough J.B. (2021). Lithium-Based Polyanion Oxide Cathodes. Nat. Energy.

[14] Yang L., Tian Y., Chen J., Gao J., Long Z., Deng W., Zou G., Hou H., Ji X. (2021). A High-Rate Capability LiFePO_4_/C Cathode Achieved by the Modulation of the Band Structures. J. Mater. Chem. A.

[15] Wang X., Yu A., Jiang T., Yuan S., Fan Q., Xu Q. (2024). Accelerating Li-Ion Diffusion in LiFePO_4_ by Polyanion Lattice Engineering. Adv. Mater..

[16] Ma J.-Q., Chen Y.-L., Peng Q., Qu Y.-P., Ding J.-F., Gong X., Yang J.-L., Qi X.-S., Zhou Y.-L. (2025). Low-Temperature Induced Crystallographic Orientation Boosting Li Storage Performance of Na_2_MoO_4_·2H_2_O. Rare Met..

[17] Tong L., Hu Z., Long Z., Tang M., Qiu X. (2023). Improvement of Electrochemical Properties of Lithium Iron Phosphate Cathode by Rare Earth Oxides Modification. J. Alloys Compd..

[18] Kim J., Song S., Lee C.S., Lee M., Bae J. (2023). Prominent Enhancement of Stability under High Current Density of LiFePO_4_-Based Multidimensional Nanocarbon Composite as Cathode for Lithium-Ion Batteries. J. Colloid. Interface Sci..

[19] Jiang X., Xin Y., He B., Zhang F., Tian H. (2024). Effect of Heteroatom Doping on Electrochemical Properties of Olivine LiFePO_4_ Cathodes for High-Performance Lithium-Ion Batteries. Materials.

[20] Zhang Q., Zhou J., Zeng G., Ren S. (2023). Effect of Lanthanum and Yttrium Doped LiFePO_4_ Cathodes on Electrochemical Performance of Lithium-Ion Battery. J. Mater. Sci..

[21] Zhou G., Wang P., Li Z., Li Y., Yao Y. (2024). Revealing Electrochemical Performance of Ni Doping LiFePO_4_ Composite. Bull. Mater. Sci..

[22] Long J., Huang J., Miao Y., Huang H., Chen X., Wu J., Li X., Chen Y. (2024). A Multi-Functional Electrolyte Additive for Fast-Charging and Flame-Retardant Lithium-Ion Batteries. J. Mater. Chem. A.

[23] Zhu R., Liu G., Qu G., Li X., Chen X., Wan W., Wang C., Huang Y. (2024). Enhancing Volumetric Energy Density of LiFePO_4_ Battery Using Liquid Metal as Conductive Agent. Adv. Funct. Mater..

[24] Liu X., Zhang Y., Meng Y., Xiao M., Kang T., Gao H., Huang L., Zhu F. (2022). Preparation and Electrochemical Properties of Co Doped Core-Shell Cathode Material on a Lithium Iron Phosphate Surface. J. Alloys Compd..

[25] Lai A., Chu Y., Jiang J., Huang Y., Hu S., Pan Q., Zheng F., Wang J., Li J., Wang H. (2022). Self-Restriction to Form *in-Situ* N,P Co-Doped Carbon-Coated LiFePO_4_ Nanocomposites for High-Performance Lithium Ion Batteries. Electrochim. Acta.

[26] Liu J., Wang S., He J., Liang K., Li J., Huang X., Ren Y. (2024). Based on N, F, and P Co-Doping Biomass Carbon to Construct 3D Porous Carbon Coated LiFePO_4_ for Preparing Lithium-Ion Batteries. J. Ind. Eng. Chem..

[27] Zhong L., Qin Y., Zhou Z., Zhong L., Liang J., Hu S., Liu Q., Zeng Y., Yi S., You H. (2025). One-Pot Formation of N, S-Doped Carbon Coated-LiFePO_4_ with Improved Lithium Storage Performance. J. Energy Storage.

[28] Han G., Hu Q., Gao K., Yao J. (2024). Boosting the Intrinsic Kinetics of Lithium Vanadium Phosphate via an Electrochemically Active Cross-Link Framework. J. Alloys Compd..

[29] Wu J., Zhong C., Chen X., Huang J. (2024). Li_3_V_2_(PO_4_)_3_ Particles Embedded in a N and S Co-Doped Porous Carbon Cathode for High Performance Lithium Storage: An Experimental and DFT Study. Inorg. Chem. Front..

[30] Huanga T., Guan L., Jin S., Zhang D., Liao W., Zhou X., Liu X., Wu C., Zeng W. (2025). Nitrogen-Doped Carbon Nanoframes Modified Li_3_V_2_(PO_4_)_3_ Derived from Phytic Acid with Ultra-High Rate Performance for Rechargeable Li Ion Batteries. Appl. Surf. Sci..

[31] Chien W., Jhang J., Wu S., Wu Z., Yang C. (2020). Preparation of LiFePO_4_/Li_3_V_2_(PO_4_)_3_/C Composite Cathode Materials and Their Electrochemical Performance Analysis. J. Alloys Compd..

[32] Chen Q., Ma G., Deng L., Ge H., Hou H. (2024). Improved Electrochemical Performances of LiFePO_4_·Li_3_V_2_(PO_4_)/C Composite Cathode Materials Prepared by Two Simple Solid-State Methods. Integr. Ferroelectr..

[33] Wei Z., Xie Z., An Y., Wu G., Zheng D., Zha J., Xiao B., Qi J., Wei F., Meng Q. (2023). 3D Network-Structured CoSb/P-CNFs@rGO as a Highly Conductive Self-Supporting Anode Material for Lithium-Ion Batteries. J. Alloys Compd..

[34] Dai H., Long Z., Li Z., Yan Z., Wang Q., Wang K., Wei Q., Qiao H. (2024). Metal-Organic Frameworks-Derived CoFe_2_O_4_/Ti_3_C_2_T_x_ MXene/Carbon Nanofibers for High-Rate Lithium-Ion Batteries. J. Alloys Compd..

[35] Su M., Lei Y., Li J., Chen Y., Niu P., Dou A., Hou X., Liu Y., Zhou Y. (2025). Electrospinning of SnSb/TiO_2_@carbon Nanofibers Anode for Lithium-Ion Batteries. Ceram. Int..

[36] Zhao W., Wang X., Wu Y., Chen S., Tao Y., Zhong Y., Zhang H., Li Y., Sun X. (2025). Multidimensionally Decorated Carbon Nanofiber through One-Step Electrospinning with Metal-Organic Framework-Derived Carbon as High-Performance Anode Materials for Lithium-Ion Batteries. J. Power Sources.

[37] Conti D.M., Urru C., Bruni G., Galinetto P., Albini B., Milanese C., Pisani S., Berbenni V., Capsoni D. (2024). Design of Na_3_MnZr(PO_4_)_3_/Carbon Nanofiber Free-Standing Cathodes for Sodium-Ion Batteries with Enhanced Electrochemical Performances through Different Electrospinning Approaches. Molecules.

[38] He Y., Pu Y., Zheng Y., Zhu B., Guo P., Zhang X., He L., Wan X., Tang H. (2024). Carbon Nanofiber-Coated MnO Composite as High-Performance Cathode Material for Aqueous Zinc-Ion Batteries. J. Phys. Chem. Solids.

[39] Zhuang H., Zhang T., Xiao H., Liang X., Zhang F., Deng J., Gao Q. (2023). 3D Free-Standing Carbon Nanofibers Modified by Lithiophilic Metals Enabling Dendrite-Free Anodes for Li Metal Batteries. Energy Environ. Mater..

[40] Liu J., Hu X., Ran F., Wang K., Dai J., Zhu X. (2023). Electrospinning-Assisted Construction of 3D LiFePO_4_@rGO/Carbon Nanofibers as Flexible Cathode to Boost the Rate Capabilities of Lithium-Ion Batteries. Ceram. Int..

[41] Zeng L., Xi H., Liu X., Zhang C. (2021). Coaxial Electrospinning Construction Si@C Core–Shell Nanofibers for Advanced Flexible Lithium-Ion Batteries. Nanomaterials.

[42] Qu H., Wei S., Guo Z. (2013). Coaxial Electrospun Nanostructures and Their Applications. J. Mater. Chem. A.

[43] Wang M., Dong X., Escobar I.C., Cheng Y.T. (2020). Lithium Ion Battery Electrodes Made Using Dimethyl Sulfoxide (DMSO)—A Green Solvent. ACS Sustain. Chem. Eng..

[44] Serrano-Garcia W., Ramakrishna S., Thomas S.W. (2022). Electrospinning Technique for Fabrication of Coaxial Nanofibers of Semiconductive Polymers. Polymers.

[45] Song W., Yang T., Shi X., Xie W., Xiao P., Lu D., Chen Y. (2025). Research on Self-Supporting Flexible Cathode Materials with High LFP Loading Based on PAN in Situ Inorganic Reaction. Mater. Lett..

